# Objective evaluation of facial flushing for mesenteric traction syndrome diagnosis during laparotomy: a prospective observational pilot study

**DOI:** 10.1186/s40981-025-00843-3

**Published:** 2026-01-06

**Authors:** Yutaro Ikki, Takeshi Negita, Kimitoshi Nishiwaki, Takahiro Tamura

**Affiliations:** 1 Department of Anesthesiology, Chitahanto Medical Center, Handa, Japan; 2 Department of Anesthesiology, Nagoya University Graduate School of Medicine, 65 Tsurumai-cho, Showa-Ku, Nagoya, 466-8550 Japan

**Keywords:** Mesenteric traction syndrome, Monitoring, Facial flushing

## Abstract

**Background:**

We aimed to evaluate the concordance between facial flushing assessment using an objective color sensor and subjective visual assessment in patients with mesenteric traction syndrome (MTS), to inform the design of a future large-scale study that incorporates objective diagnostic tools.

**Methods:**

In this prospective pilot study, we enrolled adult patients who underwent pancreatectomy. Anesthesiologists assessed the degree of facial flushing subjectively using a scale of levels 0–2. Simultaneously, the Nix color sensor objectively measured the facial redness (a-value) at four facial points. Heart rate and arterial blood pressure were also recorded. Based on the anesthesiologist’s diagnosis, patients were categorized into either the MTS or non-MTS group. We compared the ratio of changes in a-value, heart rate, and blood pressure between the two groups, and their thresholds were also calculated.

**Results:**

We included 38 patients: 14 in the MTS group and 24 in the non-MTS group. The MTS group showed significantly higher median change ratio in a-value (0.45 vs. 0.25, *P* = 0.02) and heart rate (0.45 vs. 0.23, *P* < 0.01) and a greater decrease in mean arterial pressure (-0.26 vs. -0.14, *P* = 0.01) than the non-MTS group. Subjective flushing levels correlated with MTS diagnosis (*P* < 0.01). While the Nix sensor detected flushing in some subjectively undiagnosed cases, one patient in the non-MTS group met all three objective thresholds.

**Conclusion:**

Objective evaluation of facial flushing using a color sensor is feasible and may enhance conventional assessment methods. To further improve the accuracy of objective evaluation, this pilot study may lead to a larger-scale study including blood tests.

**Supplementary Information:**

The online version contains supplementary material available at 10.1186/s40981-025-00843-3.

## Background

Mesenteric traction syndrome (MTS) is characterized by a clinical triad of hypotension, tachycardia, and facial flushing, typically triggered by surgical manipulation of the mesentery [1, 2]. MTS reportedly occurs in 30–85% of patients undergoing abdominal surgery [3–6] and is believed to result from prostacyclin (PGI_2_) release due to mesenteric traction [3].

However, the diagnosis of MTS currently relies heavily on subjective assessment, particularly visual assessment of facial flushing, which lacks standardized criteria and is prone to inter-observer variability. In this context, the development of an objective tool to quantify skin color changes could improve the consistency and accuracy of intraoperative assessment and potentially aid in resident education. Noninvasive techniques capable of detecting changes in facial blood flow or assessing the degree of redness are ideal for detecting MTS [7]. In a previous report [8], facial blood flow was continuously measured using laser speckle contrast imaging. Although this method provides an objective tool for detecting MTS, it is expensive ($58,000) ($1 = 150 JPY) and requires a considerable amount of space. Recently, the Nix color sensor (Nix Sensor Ltd., Ontario, Canada), a pocket-sized color sensor, was introduced to the market [9]. This device is cost-effective ($1,200) and easier to carry (about 200 g) than the laser speckle contrast imaging (about 2,300 g).

To our knowledge, no study, to date, has objectively quantified facial redness as a diagnostic indicator for MTS or compared it via subjective assessments. We hypothesized that quantifying color tone using the Nix color sensor would provide an objective and easy assessment for facial flushing in patients with undiagnosed MTS. Therefore, this exploratory study aimed to investigate the concordance between objective assessment using a color sensor and subjective visual assessment in determining facial flushing. Currently, the definitive diagnosis of MTS is based on the elevation of serum 6‑keto‑prostaglandin F1α (6‑keto‑PGF1α)—a stable metabolite of PGI₂. However, intraoperative measurement of this biomarker is not clinically feasible owing to its limited immediacy and laboratory dependency. Therefore, this pilot study did not aim to establish diagnostic accuracy in relation to the biochemical gold standard, but rather to explore the agreement between subjective clinical diagnosis and an objective, real-time colorimetric evaluation of facial flushing. Findings from this study may guide future large-scale studies that incorporate objective indicators into the diagnosis of MTS.

## Methods

### Design overview

This prospective, observational pilot study was conducted between June 2023 and October 2024. The study was approved by the Ethics Review Committees of Nagoya University Hospital (approval number: 2023 − 0127) and Handa City Hospital (approval number: 2024-007), and written informed consent was obtained from all participants. This study was also registered with the University Hospital Medical Research Network (approval number: UMIN000051642).

### Setting and participants

The following patients were included in the study: (i) patients aged ≥ 20 years, (ii) patients scheduled for pancreaticoduodenectomy or distal pancreatectomy for laparotomy surgery, (iii) patients with American Society of Anesthesiologists Physical Status classification of I or II, and (iv) patients who were not scheduled to be treated for MTS using non-steroidal anti-inflammatory drugs (NSAIDs) during surgery. Patients regularly using NSAIDs or antihistamines [10–12] or having a history of systemic lupus erythematosus were excluded.

### Anesthesia

Standard monitoring was implemented for all patients. In most cases, an epidural catheter was inserted between T6 and T12 vertebral levels, and a rectus sheath block and/or transversus abdominal plane block was performed in other patients. General anesthesia was induced using propofol, fentanyl, remifentanil, and rocuronium. Anesthesia was maintained with a continuous infusion of remifentanil and volatile anesthetics. Although anesthetic drugs for epidural anesthesia were used at each anesthesiologist’s discretion during surgery, epidural analgesia was initiated with a bolus injection of 3 mL of 1% lidocaine before induction of general anesthesia. Vasopressors, such as phenylephrine, ephedrine, or noradrenaline, were also administered at a mean arterial pressure of > 65 mmHg. Each anesthesiologist assessed the infusion method (bolus and continuous infusion).

### Subjective evaluation

In each patient, the anesthesiologist diagnosed MTS based on the changes in vital signs and facial flushing. Given the absence of standardized diagnostic criteria for MTS, we intentionally refrained from setting rigid definitions, aiming instead to facilitate diagnosis based on everyday clinical observations. MTS typically occurs within 30 min after skin incision, but it can also develop later. Therefore, facial flushing was assessed before skin incision and at 5, 10, 15, 20, 25, 30, 35, 40, 45, 50, 55, 60, 75, and 90 min after skin incision. The degree of facial flushing was also assessed according to a previous classification: level 0, no flushing; level 1, slight flushing; level 2, complete flushing [3, 5]. The attending anesthesiologist determined whether MTS was present and evaluated the degree of facial flushing.

Although MTS typically occurs within the first 30 min after abdominal incision, variability in surgical procedures and the timing of mesenteric traction may result in delayed onset in some cases. Therefore, we extended the observation period to 90 min after skin incision for exploratory purposes in both subjective and objective evaluations.

### Objective evaluation

**Fig. 1:**
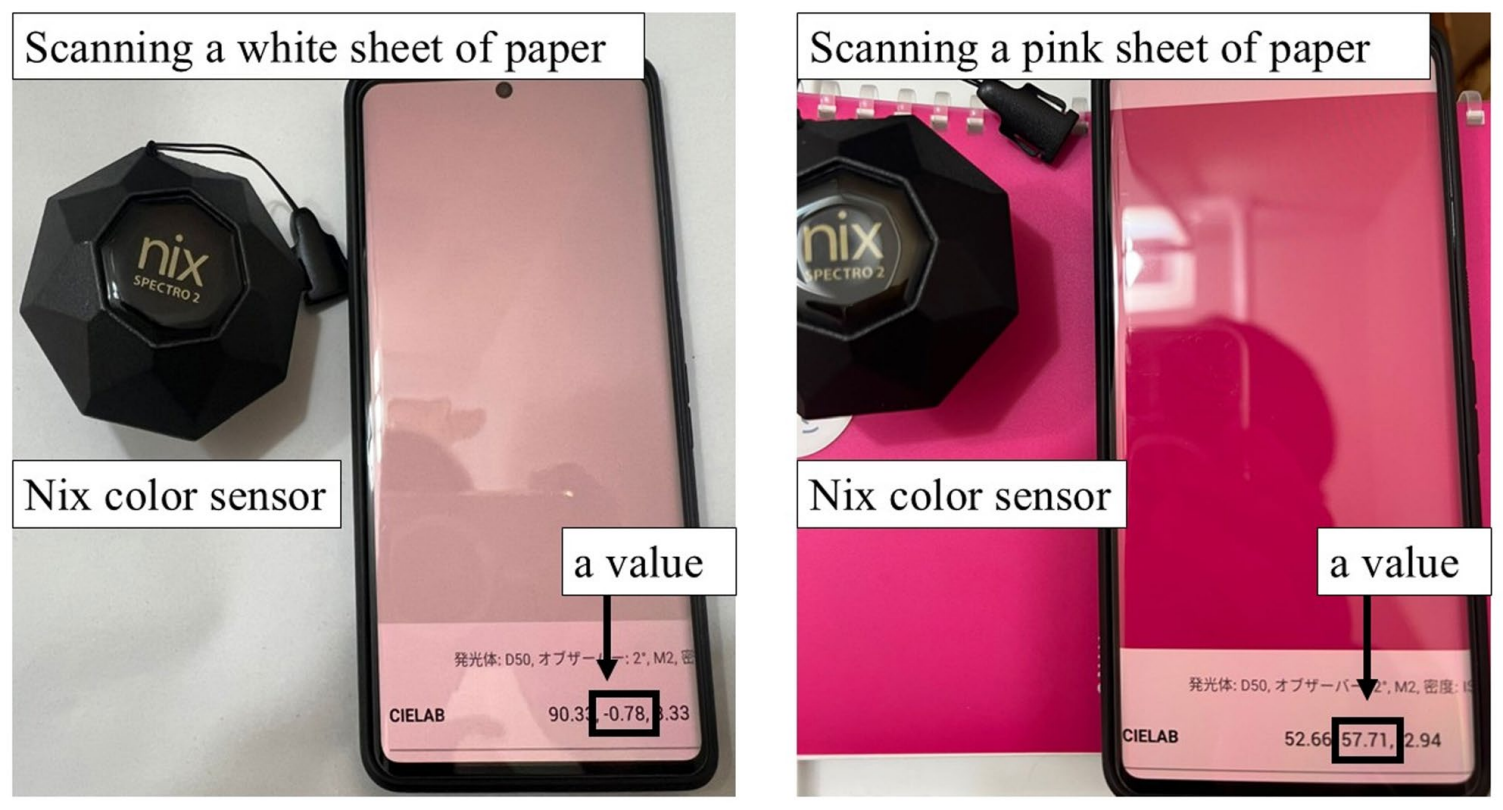
Photograph of the Nix color sensor and example scan result. Example scans of a white sheet of paper (left) and a pink surface (right). When scanned with the Nix color sensor, the a-value is displayed on a Bluetooth-connected smartphone within approximately one second

Pulse rate, arterial blood pressure and dose of vasopressor were recorded and the Nix color sensor (Nix Spectro 2, Nix Sensor Ltd., Ontario, Canada) was used to evaluate the redness of the face before skin incision and at 5, 10, 15, 20, 25, 30, 35, 40, 45, 50, 55, 60, 75, and 90 min after skin incision. The Nix Spectro 2 is a compact device measuring 6 × 6 × 5 cm and weighing approximately 200 g. Nix color sensors can be placed on any solid surface without being influenced by external light. Simultaneously, the color and different color space models were determined, including RGB, CIELab, CMYK, and HEX. This device has been successfully employed in soil, environmental, and plant sciences [9, 13]. Although it is not a medical device, its use was approved by each ethics committee. We measured facial redness (Lab color system; a-value) at four points on the face (forehead, nose, left cheek, and right cheek) as shown in Fig. 1. In the Lab color system, the range of a-values is from − 60 (green) to + 60 (red); thus, the higher the a-value, the stronger the redness.

To minimize incorporation bias, all objective colorimetric measurements were performed by an anesthesiologist whose work was independent from that of the attending anesthesiologist responsible for the subjective assessment and clinical diagnosis of MTS, ensuring parallel, independent evaluations without mutual influence.

### Classification

Participants were classified into two groups based on clinical diagnosis. Patients diagnosed with MTS by the anesthesiologist were assigned to the MTS group, while others were assigned to the non-MTS group. In the non-MTS group, we evaluated the change in a-value in patients who experienced tachycardia and decreased blood pressure with a subjective rating below level 2. The ratio of change was defined as the ratio of the difference between the maximum value (a-value and heart rate) or minimum value (blood pressure) to the base value as shown below:

a-value and HR: Ratio of change = $\:\frac{maximum\:value\:-base\:value}{base\:value\:\left(average\:of\:before\:and\:5\:min\:after\:skin\:incision\right)}$

mABP : Ratio of change =$\:\frac{minimum\:value\:-base\:value}{base\:value\:\left(average\:of\:before\:and\:5\:min\:after\:skin\:incision\right)}$

In addition, to ensure temporal alignment of the three diagnostic criteria, heart rate, mean arterial pressure, and facial a-value were analyzed using values measured at the same time point (within the same 5-min interval). Only time points where all three parameters were simultaneously available were included for calculating the rate of change. This base value was defined as the average of the values obtained 5 min before and after skin incision. Hemodynamic parameters are known to fluctuate significantly around the time of skin incision due to surgical stimulation and changes in anesthesia depth. To minimize the influence of these transient fluctuations and obtain a more stable and representative baseline, we averaged measurements from both before and after incision. In addition, at 5 min post-incision, the abdominal cavity had typically not yet been opened, meaning mesenteric traction had not occurred, supporting the validity of this time window for baseline determination. To obtain the median ratio of change, we calculated the ratios of change at four facial regions and determined their median as shown in supplementary table. The ratio of change in a-value was compared in each level because the absolute values varied significantly across cases. We also calculated thresholds for pulse rate, blood pressure, and a-value in the MTS group and then determined how many cases in the non-MTS group met those thresholds. In the MTS group, we used the values (a-value, heart rate, and mean arterial pressure) recorded at the time when all three criteria were simultaneously met and the anesthesiologist clinically diagnosed MTS. In contrast, in the non-MTS group, values showing the maximum (or minimum) deviation from baseline were used for analysis, as no diagnosis timing was available.

### Data collection

Demographic and clinical data, including sex, age, body weight, height, and body mass were obtained from the patients’ electronic medical records (HOPE/EGMAIN-GX; Fujitsu, Tokyo, Japan) and anesthesia recording system databases (ORSYS and ACSYS; Philips Healthcare, Best, Netherlands, and Prescient OR; FUJIFILM Medical Co., Ltd., Tokyo, Japan). Measurement of a-value requires approximately 1 s and enables near-simultaneous evaluation at four locations on the face.

### Statistical analysis

This was a pilot study aimed at collecting case-based data to support the design of a larger study. Based on previous reports [14], enrollment continued until 14 patients with MTS were identified. The ratios of change in a-value, heart rate, and blood pressure were compared between the MTS and non-MTS groups using the Mann–Whitney *U* test, Cochran–Armitage test, Fisher’s exact test, *t*-test, and Kruskal–Wallis test. A *P*-value < 0.05 was considered statistically significant. All statistical analyses were performed using EZR software (Saitama Medical Center, Jichi Medical University, Saitama, Japan), a graphical user interface for R (The R Foundation for Statistical Computing, Vienna, Austria) [15]. EZR is a modified version of the R Commander designed to add statistical functions that are frequently used in biostatistics.

## Results

**Fig. 2:**
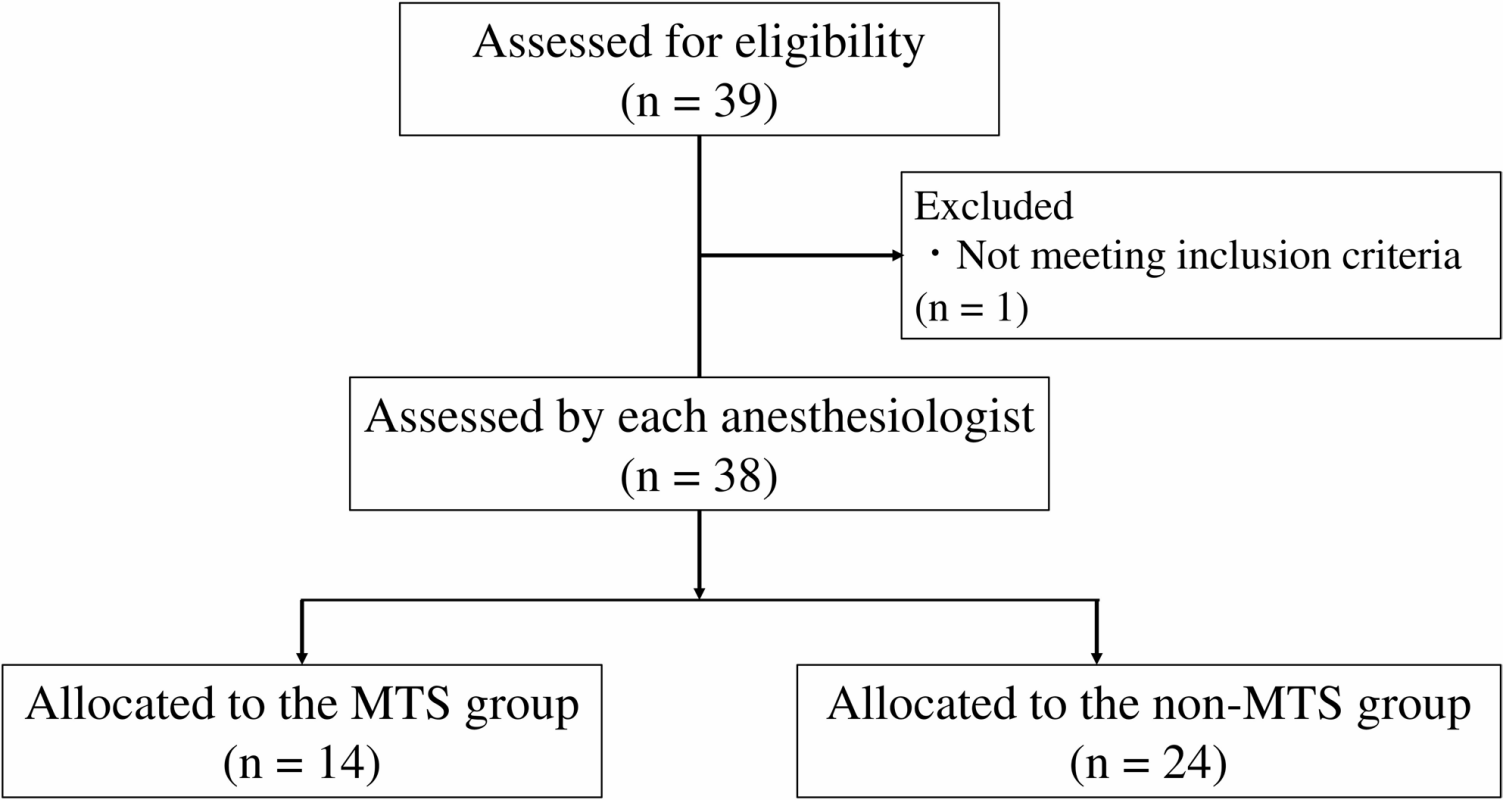
Flow diagram of included and excluded trials. Flow diagram of included and excluded trials. MTS: mesenteric traction syndrome

Thirty-nine patients were evaluated for eligibility, of whom one was excluded for taking antihistamines, and 38 were included in the final analysis. Based on subjective evaluations by the anesthesiologists, 14 patients were diagnosed with MTS and assigned to the MTS group, while 24 were assigned to the non-MTS group (Fig. 2). Patient characteristics in both groups are shown in Table 1. There were no significant differences in patient background information between the two groups, except for the dose of ephedrine administered.

**Table 1:** Background information

	MTS group	non-MTS group	*P*-value
	(*n* = 14)	(n = 24)	
Age (years)	71.1 ± 8.8	68.0 ± 10.3	0.358
Height (cm)	162.8 ± 9.4	159.7 ± 7.8	0.278
Weight (kg)	60.4 ± 11.0	55.3 ± 10.9	0.173
Male/female	4/10	14/10	0.101
Type of surgery			
PD	13	19	0.383
DP	1	5	
Type of regional anesthesia			
Epi	13	21	1
Epi + SA	0	1	
RS block and/or TAP block	1	2	
Type of vasopressor			
ephedrine (mg)	2 [0-4]	0 [0-0]	0.04
phenylephrine (mg)	0.1 [0-0.47]	0 [0-0.10]	0.23
noradrenaline (mg)	0.02 [0-0.15]	0.03 [0-0.12]	0.82

Data on age, height, and weight are shown as means ± standard deviation

Data on vasopressor dosage administered 90 min after skin incision are presented as median [interquartile range]

*MTS* mesenteric traction syndrome, *PD* pancreaticoduodenectomy, *DP* distal pancreatectomy, *Epi* epidural anesthesia, *SA* spinal anesthesia, *RS* rectus sheath, *TAP* transversus abdominal plan

P <0.05 was considered statistically significant

**Fig. 3:**
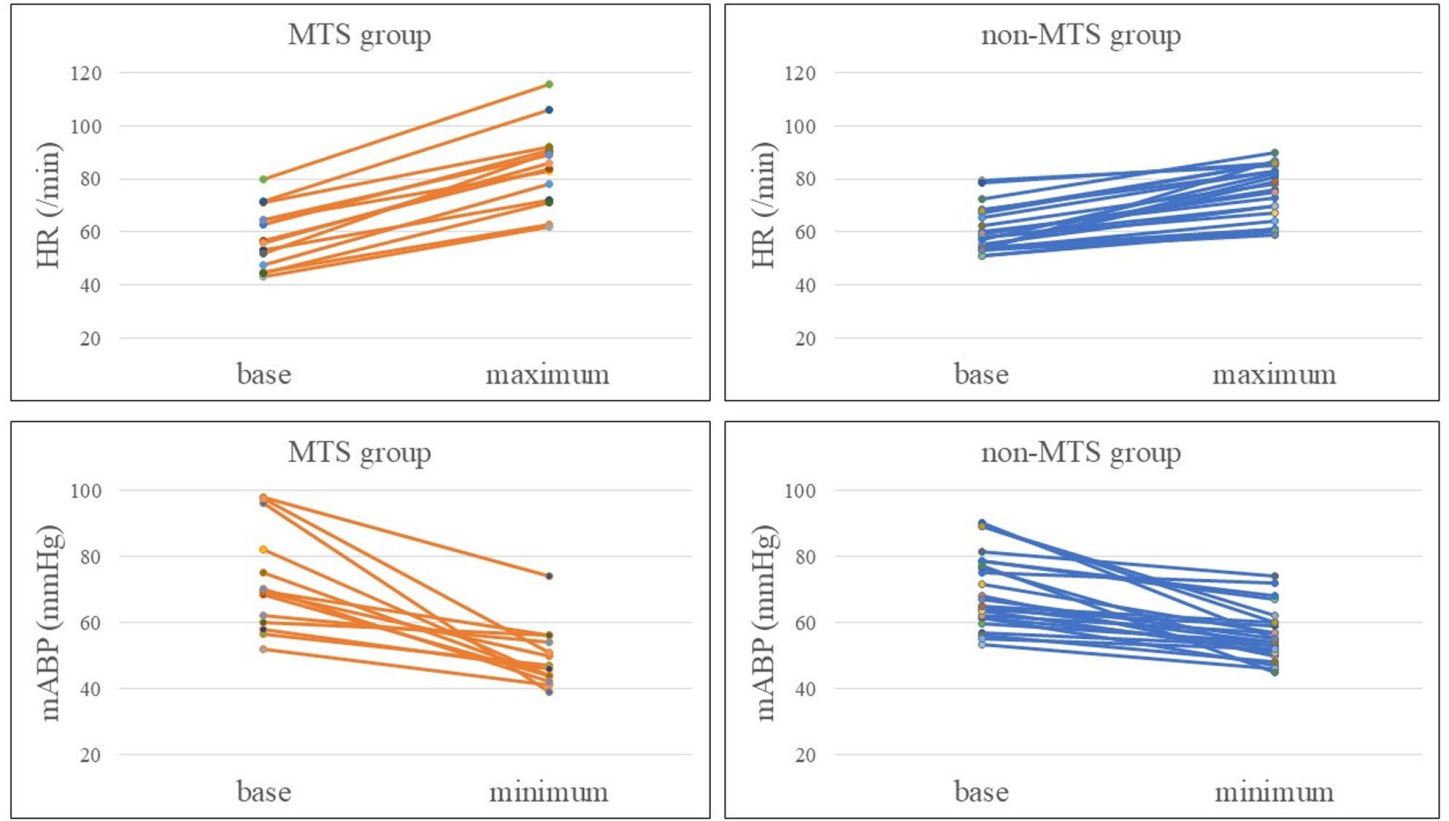
Correlation between objective and subjective evaluations. * statistically significant result.

The HR and mABP values obtained from anesthesia records in the MTS and non-MTS groups are reported in Table 2 and 3; Fig. 3. Due to substantial inter-individual variability in the a-values, absolute values were difficult to interpret and were therefore not shown here.

**Table 2:** Heart rate

	MTS group	non-MTS group	*P*-value
Base (/min)	56.3 [48.6–64.5]	58 [53.9–65.9]	0.449
Maximum (/min)	85 [73.5–90.8]	77 [66.3–83.5]	0.04
Maximum-base (/min)	27 [19.5–30.4]	14.5 [9-17.6]	<0.01

**Table 3:** Mean arterial blood pressure

	MTS group	non-MTS group	*P*-value
Base (mmHg)	69.3 [60.5–80.3]	66 [61.5–77.4]	0.639
Minimum (mmHg)	46.5 [44–53.3]	54.5 [51–60]	<0.01
Base-minimum (mmHg)	-21.5 [-30.3 to -11.3]	-10.8 [-15.4 to -5.38]	0.04

Data are presented as median [interquartile range]

*MTS* mesenteric traction syndrome, *HR* heart rate, *mABP* mean arterial blood pressure

P <0.05 was considered statistically significant

At the same time point, facial a-values were measured using the Nix color sensor. The measurement duration was approximately 1 s per site, and the error was considered minimal when measurements were taken at four points. As previously mentioned, the absolute a-values showed substantial variability across cases; therefore, for each site, the ratio of the baseline to the peak a-value was calculated, and the median of these four ratios was compared between the groups.

The median ratio of change in a-value [interquartile range] for the four facial points was 0.45 [0.43–0.49] vs. 0.25 [0.12–0.55] (*P* = 0.02) in the MTS and non-MTS group, respectively. The ratio of change in heart rate was 0.45 [0.38–0.52] vs. 0.23 [0.17–0.27] (*P* < 0.01), and the ratio of change in mean arterial blood pressure was − 0.26 [-0.41 to -0.20] vs. -0.14 [-0.24 to -0.09] (*P* = 0.01) in the MTS and non-MTS groups, respectively (Table 4).

**Table 4:** Ratio of change in each parameter

	MTS group	non-MTS group	*P*-value
a-value	0.45 [0.43–0.49]	0.25 [0.12–0.55]	0.02
HR	0.45 [0.38–0.52]	0.23 [0.17–0.27]	<0.01
mABP	-0.26 [-0.41 to -0.20]	-0.14 [-0.24 to -0.09]	0.01

Data are presented as median [interquartile range]

The ratio of change was defined as the ratio of the difference between the maximum value and baseline a-value and HR

The ratio of change was defined as the ratio of the difference between the minimum value and baseline mABP

*MTS* mesenteric traction syndrome, *HR* heart rate, *mABP* mean arterial blood pressure

P＜0.05 was considered statistically significant

The breakdown of the subjective evaluations in the MTS group was 1 for level 0, 4 for level 1, and 9 for level 2. The breakdown of the subjective evaluations in the non-MTS group was 11 for level 0, 9 for level 1, and 4 for level 2 (Table 5). As the level of subjective evaluation increased, the number of patients diagnosed with MTS also increased (*P* < 0.01).

**Table 5:** Number of cases (%) of subject assessment of facial Flushing

	MTS group (*n* = 14)		non-MTS group (*n* = 24)	
Level 0	1 (7.1%)	11 (45.8%)		
Level 1	4 (28.6%)	9 (37.5%)		
Level 2	9 (64.3%)	4 (16.7%)		

The degree of facial flushing was assessed according to previous report classification: level 0, no flushing; level 1, slight flushing; level 2, complete flushing

*MTS* mesenteric traction syndrome

**Fig. 4:**
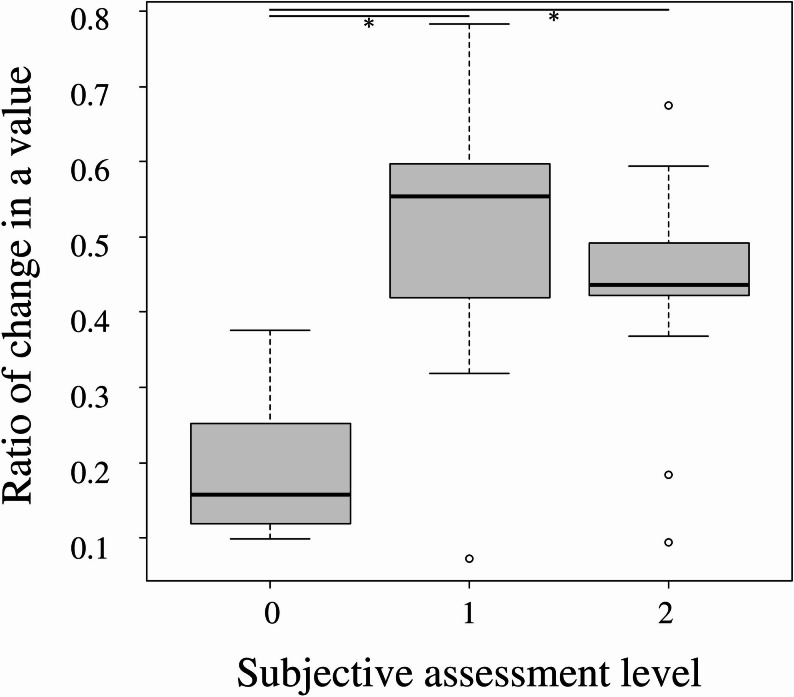
Relationship between ratio of change and subjective assessment level. * statistically significant result.

The median ratio of change in a-value was 0.158 in level 0, 0.554 in level 1, and 0.436 in level 2. The median ratio of change in a-value in level 0 was significantly lower than that in levels 1 and 2 (*P* < 0.01) (Fig. 4).

In the MTS group, the threshold values were calculated for the ratio of changes in heart rate, mean arterial pressure, and a-value. The threshold value was the sum of sensitivity and specificity, which was maximized. The threshold value of the ratio change in heart rate was 0.287 (specificity, 0.833; sensitivity, 1.000; area under the curve, 0.896; 95% confidence interval [CI], 0.791–1.0). The threshold value of the ratio change in mean arterial blood pressure was − 0.207 (specificity, 0.7; sensitivity, 0.741; area under the curve, 0.741; 95% CI, 0.57–0.912). The threshold value of the median ratio change in a-value was 0.353 (specificity, 0.538; sensitivity, 1.000; area under the curve, 0.72; 95% CI: 0.549–0.891).

Based on these thresholds, in the non-MTS group, the number of cases that met the thresholds for heart rate, mean arterial blood pressure, and a-value was five (20.8%), seven (29.1%), and 10 (41.6%), respectively. In the non-MTS group, one out of 24 cases met all the threshold criteria and had a subjective assessment below level 2.

## Discussion

This exploratory study examined for the first time differences in diagnostic agreement between objective assessment using a color sensor and subjective visual assessment of “facial flushing,” a subjective criterion among the diagnostic criteria for MTS. Two important findings were observed. First, facial flushing could be objectively evaluated by quantifying color tone using the Nix color sensor. Second, although objective evaluation of facial flushing is feasible, our findings suggest that the number of MTS cases potentially missed by subjective assessment is low. Overall, our findings indicate that the Nix color sensor performed similarly to clinical visual diagnosis and did not identify additional cases that were missed by subjective evaluation.

Several reports have suggested that the incidence of MTS ranges from 30% to 85% in patients undergoing abdominal surgery [3–6]. The proportion of patients diagnosed with MTS in our study was 36%, which lies at the lower end of this range. This variation in incidence rates across studies may be due to several factors, including differences in diagnostic criteria, timing and frequency of assessments, anesthesia protocols, and the absence of a biochemical gold standard such as 6-keto-PGF₁α, which is not available in real-time and, in some countries, is not covered by insurance, limiting its use in clinical practice. Conceivably, the use of vasopressors such as ephedrine and noradrenaline may also mask changes in vital signs, making diagnosis more difficult. Indeed, the amount of ephedrine used was higher in the MTS group than in the non-MTS group (Table 1). However, considering that the MTS group had lower blood pressure and higher a-values (Table 5), this result may be interpreted as ephedrine being administered as a treatment response to the occurrence of MTS. While remifentanil has been reported to influence the incidence of MTS [5], possibly through its sympatholytic effects that attenuate vital sign changes, previous literature suggested that NSAIDs administration was a more significant contributing factor [1, 3, 10, 11].

In our study, remifentanil was also used, but its impact appears to be limited. Given these limitations, the objective tool may not increase case detection in every context. Nonetheless, the Nix color sensor may serve as a reproducible adjunct tool that provides standardized quantification of facial flushing. This can reduce inter-observer variability, help define reference thresholds, and support multicenter trials or training in settings where diagnostic consistency is critical.

In this study, MTS was diagnosed clinically by anesthesiologists based on the presence of hypotension, tachycardia, and facial flushing, rather than by biochemical confirmation such as serum 6‑keto‑PGF1α. We acknowledge that this represents a limitation in determining the true diagnostic accuracy. Nevertheless, our findings highlight the feasibility of quantifying facial flushing as an objective, real‑time indicator that may complement biochemical validation in future studies.

Ring et al. previously reported that laser speckle contrast imaging (LSCI) could be used to quantify facial blood flow, offering an objective method for MTS detection [8]. In that study, LSCI was utilized to measure blood flow with the unit of laser speckle perfusion units (LSPU). However, LSPU reflects tissue perfusion rather than actual blood flow [16]. Although Ring et al. reported that they quantified clinical MTS based on a blood flow cutoff value, this claim appears to be an overstatement because the LSCI does not directly measure blood flow. Furthermore, a subsequent review [17] of studies on LSCI highlighted that most studies were limited to qualitative measurements and had limited interpatient comparability. Consequently, LSCI has not yet been established as a quantitative method.

Conversely, the Nix color sensor demonstrated a correlation with conventional color tones [18], suggesting its potential for objective evaluation of redness. As shown in Fig. 4, the Nix color sensor demonstrated a smaller ratio of change in a-value in cases subjectively assessed as level 0 than those assessed as level 1 and 2. Although the Nix color sensor did not directly detect the degree of facial flushing, it objectively differentiated between cases with and those without facial flushing based on subjective assessment. Thus, while it may not improve diagnostic sensitivity over current clinical practice, it offers a quantitative approach that can enhance reproducibility and transparency in research settings. Additional advantages of the Nix color sensor include its compactness, portability, and lower cost compared with the LSCI.

Unlike heart rate and blood pressure, a patient’s face cannot be monitored continuously; therefore, some cases of MTS may have been overlooked. However, in the present protocol, anesthesiologists were regularly asked to assess the degree of facial flushing, introducing a potential bias toward more frequent evaluation than usual, which may have influenced the diagnostic rate. This is precisely why objective assessment using the Nix color sensor or blood tests may offer additional potential for detecting MTS. The present results showed that the number of undiagnosed cases of MTS was not higher than expected. To further improve the accuracy of objective evaluation, this pilot study may lead to a subsequent larger-scale, multi-center study with an increased number of patients, potentially including blood tests.

### Limitation

This study has some limitations. First, the study population was limited to patients undergoing pancreaticoduodenectomy and distal pancreatectomy; thus, the generalizability of the findings to other types of abdominal surgeries remains uncertain. Second, because diagnostic criteria for MTS were not established, the time required for MTS diagnosis could not be measured. Third, although the Nix color sensor provided objective redness measurements, its performance has not been validated specifically for human skin color assessment in intraoperative settings, and factors such as skin tone, lighting conditions, and sensor pressure may have affected the readings. Additionally, this study did not include biochemical confirmation of MTS by measuring serum 6-keto-PGF1α levels. Furthermore, although subjective assessment and clinical diagnosis were performed independently, complete blinding was not feasible in the intraoperative setting. This may have introduced some incorporation bias between subjective and objective evaluations. Future prospective studies should incorporate both physiological and biochemical markers to validate the true diagnostic accuracy of objective color assessment methods. Finally, as a pilot study, the sample size was relatively small, limiting the statistical power to detect subtle differences or perform subgroup analyses. Furthermore, since other intraoperative factors such as bleeding, fluid shifts, and anesthetic depth may influence hemodynamics over time, interpretation of data acquired later in surgery (e.g., after 45 min) may be affected by these confounders.

## Conclusion

Facial flushing in MTS can be objectively assessed by quantifying color tone using the Nix color sensor. To achieve a more objective diagnosis of MTS, larger-scale studies will be necessary in the future, and the Nix Color Sensor may serve as a supplementary tool in this process.

## Supplementary Information

Supplementary Material 1.

## Data Availability

The datasets used and analyzed in this study are available from the corresponding author on reasonable request.

## Abbreviations

CI: Confidence interval
HR: Heart rate
LSCI: Laser speckle contrast imaging
LSPU: Laser speckle perfusion units
mABP: mean Arterial blood pressure
MTS: Mesenteric traction syndrome
NSAIDs: Non-steroidal anti-inflammatory drugs
PGI_2_: Prostacyclin
