# Supplementary material for: Objective evaluation of facial flushing for mesenteric traction syndrome diagnosis during laparotomy: a prospective observational pilot study
Source: JA Clin Rep. 2026 Jan 6;12:6. doi: 10.1186/s40981-025-00843-3 (PMC12783494; doi:10.1186/s40981-025-00843-3)
Supplement: Supplementary file 1 — Supplementary Material 1. [file 40981_2025_843_MOESM1_ESM.docx]

**Supplementary Table. Method for Calculating Median Percent Change and Variable Definitions**

| **Case 1** | **Forehead** | **Nose** | **Left cheek** | **Right Cheek** |
| --- | --- | --- | --- | --- |
| **Base value** | 〇 | △ | □ | ◇ |
| **Maximum value** | ● | ▲ | ■ | **◆** |
| **Max-baseline** | ●-〇 | ▲-△ | ■-□ | **◆**-◇ |
| **The Ratio of change =**$\frac{\boldsymbol{maximum} \boldsymbol{value} \boldsymbol{-} \boldsymbol{base} \boldsymbol{value}}{\boldsymbol{base} \boldsymbol{value}}$ | $\frac{●-〇}{〇}$ | $\frac{▲-\triangle}{\triangle}$ | $\frac{■-\square}{\square}$ | $\frac{\mathbf{◆}-◇}{◇}$ |

The median ratio of change is defined as the ratio of change across the four facial regions in Case 1. In Case2-38, the median ratio of change was calculated using the same method as in Case1. The 38 cases were divided into the MTS and non-MTS groups, and the median of the median ratio of change was calculated for each group. A comparative analysis was then conducted between the two groups.
